# Linear approximation of variable relative biological effectiveness models for proton therapy

**DOI:** 10.1016/j.phro.2024.100691

**Published:** 2024-12-24

**Authors:** Dirk Wagenaar, Johannes A. Langendijk, Stefan Both

**Affiliations:** Department of Radiation Oncology, University Medical Center Groningen, University of Groningen, Groningen, the Netherlands

**Keywords:** Relative Biological Effectiveness (RBE), Linear Energy Transfer (LET), Proton therapy

## Abstract

The McNamara (MCN) and Wedenberg (WED) RBE weighted dose (D_RBE_), dose and dose-weighted average LET (LET_d_) were calculated in twenty brain cancer patients. A linear approximation was made for each RBE model to give best agreement to clinically relevant dosimetric parameters. Additional evaluations were done on twenty head and neck and twenty breast cancer patients.The R^2^ of the fits was ≥0.94 and ≥0.91 for MCN and WED respectively for α/β values ≥1.0 Gy. The graphs derived in this work can be used to convert RBE-LET slopes derived from clinical data to α/β values in the MCN or WED models.

## Highlights

• The McNamara and Wedenberg models have been simplified to linear approximations.
• This was fit was done using clinically relevant endpoints.
• These approximations can be used to compare clinical results to existing RBE models.

## 1 Introduction

The relative biological effectiveness (RBE) factor converts proton dose to its equivalent photon dose known as the RBE weighted dose (D_RBE_) with unit Gy_RBE_ [1], [2]. The RBE of protons is taken as a constant for clinical use, but preclinical evidence suggests a variable RBE depending on the dose-weighted average linear energy transfer (LET_d_), fraction dose and the α/β [3], [4], [5], [6], [7], [8]. Of these factors, the increase of RBE with LET_d_ is of particular interest as this leads to a potential increase in D_RBE_ at the end of proton range which is sometimes called the end-of-range effect or RBE effect [7]. Protons often stop in or near organs-at-risk (OARs), leading to a clinical need to understand the RBE-LET_d_ relation so that high biological effect in the OARs can be prevented, in particular in serial structures [7], [9], [10], [11], [12], [13], [14].

Different RBE models exist with varying levels of complexity [4]. A constant RBE of 1.1 is advised for tumor prescription in clinical practice [1]. Linear models increase RBE with increasing LET_d_ and serve as a first-order approximation [15], [16]. Phenomenological models based on the linear-quadratic model for cell survival seek to incorporate the effects of fraction dose and the endpoint specific α/β value and have a nonlinear relation with LET_d_ [5], [6].

Phenomenological are commonly derived from preclinical data and such models are starting to see clinical adoption [17]. However when analyzing clinical data, RBE models are often inferred with a linear relationship with LET_d_ from logistical regression results [9], [18], [19]. Additionally, when tools for LET_d_ optimization become available, these might not be able to optimize D_RBE_ for the RBE model of choice directly, creating a need for the conversion of phenomenological RBE models to a linear equivalent [15].

Therefore, the aim of this study was to make linear approximations and quantify the non-linearity of two commonly used RBE models for various α/β values.

## 2 Materials and methods

This study was conducted in three sets of 20 consecutive adult brain, head & neck and breast cancer patients treated with proton therapy resulting in a total sample of 60 patients. All patients started treatment in 2019 and finished before November 2019. All patients gave written informed consent for scientific use of their data.

Patients were treated with proton pencil beam scanning delivered with a Proteus ®Plus (IBA, Ottignies-Louvain-la-Neuve, Belgium). The treatment plans were generated using robust optimization in the treatment planning system (TPS) RayStation v9A (RaySearch Laboratories, Stockholm, Sweden) with a 3 % range uncertainty setting and a site specific setup uncertainty setting. Target coverage was assessed using the voxel-wise minimum robustness evaluation adopted by all Dutch proton centers, with the criteria that 95 % of all target volumes should receive at least 98 % of the prescribed dose (i.e. V_95%_ > 98 % and D_98%_ > 95 %) in the voxel-wise minimum of all robustness scenarios [20]. Target prescriptions are defined assuming a constant RBE of 1.1.

Brain cases were prescribed 28, 30 or 33 fractions of 1.8 Gy_RBE_. Brain cases were planned with two to four beams with a single field uniform dose (SFUD) technique to limit plan modulation. HNC patients were prescribed a total dose of 70 Gy_RBE_ to the primary and 54.25 Gy_RBE_ to the prophylactic CTV in 35 fractions. Treatment planning for HNC patients was done with an intensity modulated proton therapy (IMPT) technique with up to six beams from 4 directions with and without range shifters [[21], [22]]. Breast and thoracic wall patients were prescribed 15 or 20 fractions of 2.67 Gy_RBE_. Treatment planning for these cases was typically done using one or two IMPT beams but up to four beams were used if necessary and beams were delivered with a range shifter and five times volume repainting to reduce interplay effects.

All clinically used treatment plans were exported to a development build of RayStation based on v9R which enables LET_d_ and D_RBE_ calculations from the Monte Carlo dose calculation [23]. Here, the voxel-wise product of dose and LET_d_ (D⋅LET_d_) and the D_RBE_ for the McNamara (MCN) and Wedenberg (WED) RBE models using an α/β value between 0.2 and 10.0 Gy were calculated in steps of 0.2 Gy [5], [6]. The MCN and WED models were used as they are developed independently and are frequently used in recent publications investigating potential RBE effects [[4], [24], [25], [26], [27], [28], [29]]. The dose, D·LET_d_ D_RBE_ of the MCN and WED models and voxel volumes of all considered regions-of-interest (ROIs) were exported for subsequent analysis outside the treatment planning system.

Linear approximations of each RBE model were fitted using dosimetric parameters from brain patients, as variable RBE is considered to be most concerning in this body site. The considered parameters were the near maximum D_RBE_ to at least 0.03 cc (D_0.03cc_) of the brainstem, optic nerve left and right and chiasm and to at least 3.00 cc (D_3cc_) of the brain, resulting in five parameters per patient [30].

For finding the slope best describing each RBE model, a least squares fit was made by minimizing the following loss function:

$$f={\sum_{i}\left(D_{i,vRBE}-D_{\begin{array}{c} i,linear \end{array}}\left(c\right)\right)}^{2}$$

In which the *i* are the different dosimetric parameters, *D_i, vRBE_* is the dosimetric parameter calculated with MCN or WED, *Di, linear(c)* is the dosimetric parameter calculated with a linear model of the form $\mathrm{RBE}=1.0+c∙LET_{d}$. Dosimetric parameters were calculated in Python and the SciPy Python module was used to find the slope c which minimizes the loss function [31]. The calculation of dosimetric parameters in Python was validated by cross-checking with values calculated in the TPS. Separate fits were made for both the MCN and WED models and all α/β values.

The agreement of fit was determined using the coefficient of determination (R^2^). The R^2^ was calculated by comparing the additional biological dose (i.e. D_RBE_-D) for both the variable RBE model and its linear approximation. The D_RBE_-D was used instead of the D_RBE_ as it is much more sensitive to differences (i.e. the R^2^ is lower). The linear approximations were also applied to organ-at-risk D_RBE_ calculations of twenty head and neck and breast cancer patients to investigate the fit agreement in different body sites and prescriptions. Parameters used for assessing linear model agreement in head and neck cancer patients were the mean dose to the parotid glands, submandibular glands, pharyngeal constrictor muscle superior, mid and inferior, cricopharyngeal muscle, supraglottic, oral cavity, glottic area and the near maximum dose D_0.03cc_ to the thyroid, spinal cord and brainstem (14 parameters) and for breast cancer patients the mean dose to the esophagus, heart, lung, thyroid, and breasts (6 parameters).

## 3 Results

**Fig. 1:**
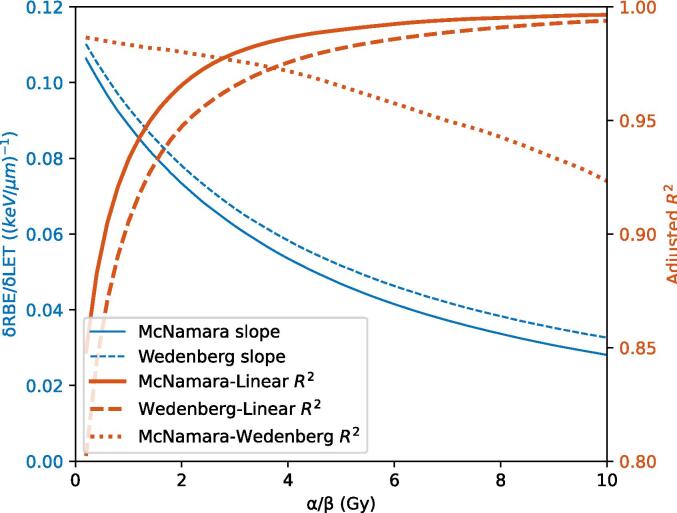
Slope of the RBE-LET_d_ relation and coefficient of determination (R^2^) as a function of α/β. R^2^ calculated using the difference with physical dose (i.e. D_RBE_ – D_phys_).

The slope varied between 0.11 and 0.03 (keV/μm)^−1^ for the MCN model and between 0.11 and 0.03 (keV/μm)^−1^ for the WED model (Fig. 1). The R^2^ was between 0.85 and 1.00, and between 0.80 and 0.99 for the MCN and WED models respectively, with a value of 0.97 and 0.95 for an α/β of 2.0 Gy respectively. The R^2^ increased with increasing α/β and was higher for the MCN than WED models, indicating that the RBE models behave more linearly for higher α/β values. For an α/β of 2.8 or higher, the agreement between the MCN and its linear estimate was higher than the agreement between the MCN and WED models.

**Fig. 2:**
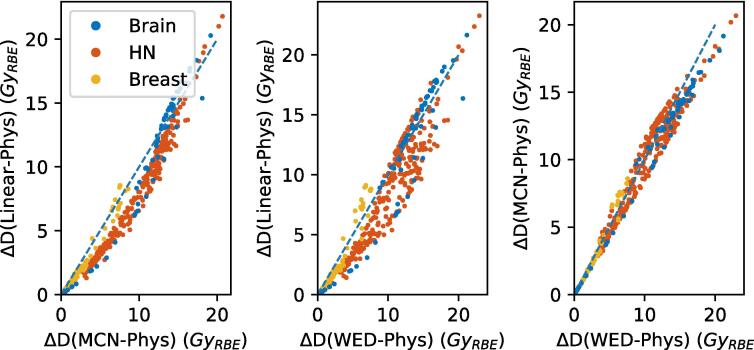
Calibration curves of the McNamara, Wedenberg and linear models. Each datapoint represents a dosimetric parameter tied to a clinical goal. An α/β of 2.0 Gy was used. The figures illustrate the disagreement between McNamara and a linear fit (left), Wedenberg and a linear fit (middle) and between McNamara and Wedenberg. For perfect agreement between models all points are expected to lie on the dotted line. The linear fits were made based on parameters of brain patients (blue), but also head and neck (red) and breast (orange) patients are shown. (For interpretation of the references to colour in this figure legend, the reader is referred to the web version of this article.)

When applying the linear approximations to the head and neck and breast cancer cohorts (Fig. 2) assuming an α/β of 2.0 Gy, the R^2^ decreased from 0.97 to 0.80 and 0.97 respectively for the MCN model and from 0.95 to 0.77 and 0.92 respectively for the WED model. The mean absolute error was 1.22 Gy_RBE_ and 1.33 Gy_RBE_ across all body sites with a 95th percentile near max error of 2.95 Gy_RBE_ and 4.06 Gy_RBE_ respectively for the MCN and WED models respectively. Overall, the linear approximations can be observed to slightly underestimate the DRBE in the low dose region, while overestimating the dose in the high dose region (Fig. 2).

## 4 Discussion

Linear approximations were made fitted to clinically relevant dosimetric parameters for neuro-oncological patients. For a commonly used α/β value of 2.0 Gy, the agreement was very good with an agreement of over 95 % of variance in additional biological dose (R^2^ > 0.950). The agreement increased for increasing α/β with a better agreement between the variable RBE model and their linear approximation than the agreement between both variable RBE models for an α/β > 2.8 Gy.

Bahn et al. made an NTCP model for the local risk of contrast-enhancing brain lesions after proton therapy and found an RBE-LET slope of 0.10 (keV/μm)^−1^ [9]. They indicated this RBE model with a linear slope was like the WED model with an α/β of 2.0 Gy. Based on the results of our study their linear RBE model more closely resembles the MCN or WED model with an α/β of 0.6 Gy which also have a slope of 0.10 (keV/μm)^−1^ [18]. Using an α/β of 2.0 Gy instead would result in RBE values that are 0.11 higher in the high LET region of 5.0 keV/μm. It is worth noting that the RBE-LET slope derived from clinical data does not necessarily fall in the range of slopes for these RBE models. For instance, Eulitz et al. derived an RBE-LET slope of 0.12 (keV/μm)^−1^ analyzing the incidence of brain lesions after proton therapy. There is no α/β value for which the MCN or WED models have the same approximate result.

The concept of simplifying variable RBE models to a linear equivalent might be appealing as a first-order approximation of a concept with many uncertainties [4], [8]. Our analysis showed that the linear approximations are very similar to the variable RBE models for a fixed α/β value. Even so, errors in the order of 2.9–4.1 Gy_RBE_ can still occur. Such errors tend to be overestimations in low-dose regions or overestimations in high-dose regions as the variable RBE models take into account an increasing RBE with decreasing fraction dose. It should be noted that in our analysis the α/β value was assumed to be known, while in reality the uncertainty in α/β value is a major concern in RBE uncertainty as shown by McMahon et al. [8].

The purpose of integrating LET optimization into treatment planning is to reduce the normal tissue complication probability (NTCP) by reducing D_RBE_ in the OARs and/or increasing tumor control probability (TCP) by increasing the D_RBE_ in the target [[15], [32]]. The RBE-LET relation is uncertain due to differences in LET_d_ definition, RBE model definition and outcome to which the models were fitted [[4], [33], [34]]. Therefore, using the D·LET_d_ in LET optimization is an effective way to reduce the mean D_RBE_ of OARs. However, it might be less helpful in trying to reduce the maximum D_RBE_ as the highest D·LET_d_ value might occur in another location than the maximum D. Similarly, using D·LET_d_ is suitable when trying to relate patient toxicity to LET_d_ for parallel OARs[9], [18].

An important limitation of our work is that we only investigated two RBE models, namely the MCN and WED models which are used in many other studies [[4], [24], [25], [26], [27], [28], [29]]. Previous work focused on a review of a larger number of RBE models but evaluated these in a more limited setting using fewer patients and dosimetric parameters. Additionally, our results were tested in only three treatment sites. The considered treatment sites are very different in anatomy and beam setup and the results were not very different among the considered sites. Still, the results could be different for prescribed fraction doses outside the range of 1.80 and 2.67 Gy_RBE_.

The graphs derived in this work can be used to convert RBE-LET slopes derived from clinical data to α/β values in the MCN or WED models. Assuming a linear RBE-LET_d_ relation shows good overall agreement but can in some cases introduce uncertainties in clinically relevant parameters in the order of magnitude of up to 4.0 Gy_RBE_. However, this uncertainty might be acceptable when compared to differences between variable RBE models. A linear RBE approximation can be used when relating clinical endpoints to D and D⋅LET_d_ independently. However, caution is warranted when evaluating the near maximum dose to critical organs-at-risk for clinical practice using a linear RBE approximation.

## CRediT authorship contribution statement

**Dirk Wagenaar:** Conceptualization, Data curation, Formal analysis, Investigation, Methodology, Visualization, Writing – original draft, Writing – review & editing. **Johannes A. Langendijk:** Conceptualization, Funding acquisition, Supervision, Writing – review & editing. **Stefan Both:** Conceptualization, Funding acquisition, Methodology, Supervision, Writing – review & editing.

## Declaration of competing interest

The authors declare that they have no known competing financial interests or personal relationships that could have appeared to influence the work reported in this paper.
